# Patient Safety and Quality of Care are Everybody's Business: Evaluating the Impact of a Continuing Professional Development Program beyond Satisfaction

**DOI:** 10.15694/mep.2019.000046.1

**Published:** 2019-03-11

**Authors:** Francesca Luconi, Miriam Boillat, Suzanne Mak, Daniel Chartrand, Nadine Korah, Mark Daly, Meron Teferra, Jennifer Gutberg

**Affiliations:** 1 McGill University; 2 University of Toronto

**Keywords:** Patient safety, quality improvement, continuing professional development (CPD), knowledge translation, outcome-based evaluation, interprofessional

## Abstract

This article was migrated. The article was marked as recommended.

Background

Research integrating Continuing Professional Development (CPD) with patient safety (PS) and quality improvement (QI) is still in its infancy despite advocacy by leaders in the field.

Objectives

This theory-driven study explored the feasibility to implement and evaluate the impact of a CPD intervention focused on teaching and practicing PS and QI at the levels of satisfaction, usefulness, knowledge, confidence, intention to change behaviour and reported changes in practice.

Methods

Three workshops targeting healthcare professionals were delivered live between 2014 and 2016. Data was collected longitudinally through four questionnaires and analyzed with descriptive statistics and triangulation of sources. Thematic analysis of qualitative data was guided by the Theoretical Domains Framework.

Results

Sixty-seven healthcare professionals participated in the study. Across workshops, satisfaction was high and a significant increase in knowledge and confidence were reported immediately post-intervention. Intention to change behavior was high across workshops. *‘Moral norm’* and ‘ *beliefs about consequences’* were consistently rated as the most influential factors in participants’ intention to change behavior while ‘ *social influence’* was consistently rated as the least influential. At the workshops, participants anticipated improving communication, increasing their knowledge on PS-QI, applying content learned and building teamwork. Commonly anticipated barriers to implementation included lack of resources, environmental stressors, and the organizational climate/culture. These barriers were confirmed six-month post where participants reported partially implementing 78% (18/23) anticipated goals.

Conclusions

This study showed the feasibility to develop and implement an effective CPD intervention supporting healthcare professionals’ knowledge, confidence, and reported change in teaching and practicing PS-QI.

## Introduction

Patient safety (PS) as a discipline moved into the spotlight in 1999, when the Institute of Medicine published its landmark study, suggesting that up to 100,000 patients died due to adverse events (AE) ( Kohn, Corrigan and Donaldson, 2002). A comprehensive widespread reform of the health system to improve quality of care has been advocated following the model proposed by the Institute of Medicine that characterized quality of care as: safe, timely, effective, efficient, equitable and patient-centered ( Institute of Medicine, 2001). Though great efforts have been made to improve healthcare systems since then, evidence surrounding PS outcomes has not followed suit, as indicated by a recent estimate of medical error as the third leading cause of death in the United States. ( Makary and Daniel, 2016; Davis and Rayburn, 2016).

Patient safety as a core competency is woven throughout the competency-based medical education (CBME) model, which has been gradually adopted in undergraduate and post-graduate medical curricula in Canada (Ginsburg, Tregunno and Norton, 2013; Potts, Shields, and Upshur, 2016) and internationally ( Miller and Hoffman, 2005) and is defined as “an outcome-based approach to the design, implementation, assessment and evaluation of a medical education program using competencies as the organizing framework” ( Frank *et al*., 2010). PS has been defined as part of, or directly related to quality improvement (QI), ( Davis and Rayburn, 2016; Kitto *et al.,* 2015) which is equally recognized as a core component of CBME.

The teaching and practicing of PS and QI have been investigated worldwide ( Altin et al., 2014; Czabanowska et al., 2012; Bethune *et al.,* 2013; El-Jardali, and Fadlallah, 2017). In Canada, competency-based continuing professional development (CB-CPD) is planned to start in 2020 and is rooted in CBME, life-long learning and the revised CanMEDS ( Frank, Snell, and Sherbino, 2015) and the CanMEDS-Family Medicine Framework ( The College of Family Physicians of Canada, 2017). According to the 2015 CanMEDS framework, “A key competency addresses the evolving recognition of patient safety and continuous quality improvement as important components of medical expertise at the bedside” ( Frank, Snell, and Sherbino, 2015, pp. 10).

Continuing professional development (CPD) has been suggested as an effective Knowledge Translation (KT) strategy for improving healthcare professionals performance, patient outcomes, (Ginsburg, Tregunno and Norton, 2013; Cervero and Gaines, 2015) driving system change ( Davis and Rayburn, 2016; Kitto *et al.,* 2015; Sklar, 2016) and changing the system’s culture ( Miller and Hoffman, 2005). However, CB-CPD must adopt a different ‘paradigm’ than traditional CPD, and must ultimately target clinical outcomes ( Van Hoof, and Meehan, 2011)in alignment with PS and QI ( Davis *et al.,* 2013) and CBME ( Sargeant, Wong and Campbell, 2018). Research integrating CPD with PS and QI is still in its infancy despite advocacy by CPD leaders ( Davis and Rayburn, 2016; Kitto *et al.,* 2015; Davis *et al.,* 2013; Batalden and Davidoff, 2007) accrediting and licencing bodies ( Lemire, 2016) and specialized institutions ( Sklar, 2016; Canadian Patient Safety Institute, 2018; Canadian Medical Protective Association, 2018). Unfortunately, the majority of CPD activities are still not being designed to meet this paradigm shift, nor are they based on outcome-based theoretical frameworks ( Moore, Green and Gallis, 2009). In fact, the majority of accredited CPD activities target ‘cognitive’ domains focused on demonstration of knowledge acquisition, rather than measurable performance change ( Légaré *et al.,* 2014). Theory-based PS-QI initiatives outside of the CPD literature have been linked to performance change, such as clinical outcomes in diabetes care ( Doyle *et al.,* 2014).

Traditional conferences and workshops only increase knowledge and practice behaviours ( Chipchase, Johnston and Long, 2012; Forsetlund *et al, 2009*) whereas combining interactive and didactic formats might influence patient outcomes. There is a lack of studies focused on longitudinal, classroom training to teach general PS-QI principles to multi-professional teams ( Rabol, Ostergaard and Mogensen, 2010). Furthermore, there is scarcity of theory-driven educational interventions focused on PS-QI. Wallace et al., (2009) using the Theory of Planned Behavior ( Ajzen, 1991)developed a training program on root cause analysis. An intervention focused on aggression management training ( Oostrom and van Mierlo, 2008), guided by the Kirkpatrick evaluation model ( Kirkpatrick, 1975) reported solely behavioral outcomes. Beyond these examples, the existing literature on PS interventions in CPD offers scarce evidence of systematically evaluated activities that not only consider behavioral performance outcomes but specifically those that relate to patients’ outcomes ( Leifso, 2014; Shah, Cross and Sii, 2013; Stevens, 2011; Dauer *et al.,* 2006),with fewer still utilizing an outcome-based theory-driven approach. Furthermore, there is a need to change the culture within the workplace to address the growing gap between the formal and informal curricula on PS and QI ( Martinez *et al.,* 2014). This need is well-recognized, and as Law *et al.* suggest: “in order to improve safety culture, it is essential to base changes on a framework of safety culture” (2010, pg. 110). The present study takes an important step in creating culture change by taking an explicitly framework-based, theory-driven approach that further addresses the fundamental tenets of safety culture, including teamwork, open communication, and blame-free environments to support learning ( Kenneth Milne *et al.,* 2010; Boaro, 2010).Trained clinical teachers and practitioners could serve as powerful role models to bridge this gap and enhance feasibility and implementation of PS practice changes ( Soo, Berta, and Baker, 2009; Wakefield *et al.,* 2010; Baum and Davis, 2017).

The present study adopted an integrated approach to CPD in order to address the existing gaps in the literature and in practice ( Gagnon *et al.,* 2003) and is driven by a theoretical framework that combines principles from socio-cognitive theories ( Légaré *et al.,* 2014; Slotnick, 2001), the Theory of Planned Behaviour ( Ajzen, 1991), the Outcome-Based Evaluation framework ( Davis *et al.,* 2013) and Knowledge-to-Action framework ( Graham *et al.,* 2006). It represents the first initiative to bridge the silos amongst the CPD office, affiliated teaching hospitals and Faculty Development, viewed as a force to promote organizational change ( Baum and Davis, 2017).

This study is driven by the following research questions: (1) To what extent is it feasible to develop, implement and evaluate the impact of an accredited CPD intervention focused on PS and QI? (2) What are the participants’ knowledge and confidence gaps prior to and immediately after the intervention? (3) What are the attendees’ perceptions of the impact of the intervention on their ability to teach and/or practice PS-QI? (4) What is the 6-month reported impact of the intervention in clinical and/or teaching practices?

## Methods

We conducted a longitudinal, theory-driven program evaluation study that assessed feasibility, participation, satisfaction, knowledge, and self-reported performance outcomes. This study was approved by the Institutional Review Board (IRB) of a Canadian university. The interprofessional planning committee, composed of local champions in PS-QI, CPD and faculty development led the creation of the certified CPD intervention. Triangulation of sources from a content-specific needs assessment informed the design and implementation of the intervention in alignment with the six competencies from the Canadian Patient Safety Institute ( Table 1) ( Canadian Patient Safety Institute, 2018) and CanMEDS frameworks ( Frank, Snell, and Sherbino, 2015; The College of Family Physicians of Canada, 2017).

**Table 1.** Canadian Patient Safety Institute: Six domains of competencies

Contribute to a Culture of Safety
Work in Teams for Patient Safety
Communicate Effectively for Patient Safety
Manage Safety Risks
Optimize Human and Environmental Factors
Recognize, Respond to and Disclose Adverse Events

The intervention covered three consecutives certified workshops for healthcare professionals. Each workshop addressed specific learning objectives, competencies and instructional strategies and included plenaries followed by moderated practice-based small group discussions ( Table 2). With the exception of Workshop I (repeated) which was delivered in French, all workshops were delivered in English by PS-QI educators.

**Table 2.** CPD intervention: Patient Safety Workshop Series

	Date	Site	Learning objectives	Target audience
**Workshop I**	September 18, 2014	University Quebec, Canada	Patient safety principles; instructional strategies, disclosure process and importance of role modeling.	Undergraduate and postgraduate program directors and site directors
**Workshop I (repeated)**	September 24, 2015	University Quebec, Canada	Patient safety principles; instructional strategies, disclosure process and importance of role modeling.	Healthcare professionals working at teaching hospitals and other sites.
**Workshop II**	November 9, 2015	Teaching hospital, Quebec, Canada	Team communication: Situation-Background-Assessment-Recommendation (SBAR); Managing High Risk Situations covered Transitions and Handovers.	Healthcare professionals working at teaching hospitals and other sites.
**Workshop III**	November 7, 2016	Teaching hospital, Quebec, Canada	Strategies to embed QI in clinical practice and education. Discussion of barriers to implementation and how to overcome them.	Healthcare professionals working at the teaching hospital and other sites.

Prior to the workshops, attendees were contacted with an invitation to participate in the research study. All workshop attendees, regardless of their participation in the study, received the same workshop content and all completed the Program Evaluation. The effectiveness of the CPD intervention was assessed via six outcomes and five measures ( Table 3).

**Table 3.** Alignment of research questions with outcomes and data sources

Research question	Outcome	Data sources
To what extent is it feasible to develop implement and evaluate the impact of an accredited CPD intervention focused on PS and QI?	Participation Usefulness Satisfaction	Attendance records and drop-out rates Program Evaluation
What are the participants’ knowledge and confidence gaps prior to and immediately after the intervention?	Knowledge Confidence	Post-Workshop Retrospective Pre-Post questionnaire
What are the attendees’ perceptions of the impact of the intervention on their ability to teach and/or practice PS-QI?	Expected/intended performance	CPD-Reaction Questionnaire (Intention to change practice)Personal Action Plan (I)
What is the 6-month reported impact of the intervention in clinical and/or teaching practices?	Reported performance	Personal Action Plan (II)

### Outcome Measures

Participants completed four outcome measures ( Table 3) immediately after each workshop. The Post-Workshop Retrospective Pre-Post Questionnaire (Supplementary File 1) measured participants’ perceived knowledge and confidence levels on 8 statements derived from the learning objectives. The Program Evaluation (10-item questionnaire), measured satisfaction and the usefulness of the workshops. The CPD-Reaction questionnaire (Supplementary File 3) is a validated, theory-based 12-item instrument consisting of five constructs, and evaluates intention to change a specific behavior derived from one of the workshop’s learning objectives ( Table 4) ( Légaré *et al.,* 2011; Légaré *et al.,* 2014; Légaré *et al.,* 2017). The specific behaviors included: “ *Apply the disclosure guidelines to my practice”* (W1 and W1-R); *“Apply the Situation-Background-Assessment-Recommendation (SBAR) to my practice”* (W2); “ *Apply QI strategies to solve challenges in my practice”* (W3).

**Table 4.** CPD-Reaction questionnaire scores on items and constructs

Construct scale	Definition	Items	Response Choices
Intention	An individual’s motivation to adopt a specific behavior or not	I intend to *[behaviour]*	Strongly disagree/agree
		I plan to *[behaviour]*	Strongly disagree/agree
Social Influence	Perception of approval or disapproval by persons significant to the individual regarding the adoption of the behavior	To the best of my knowledge, the percentage of my colleagues who *[behaviour]* is...	0-20% 21-40% 41-60% 61-80% 81-100%
		Now think about a co-worker whom you respect as a professional. In your opinion, does he/she *[behaviour]*	Never/Always
		Most people who are important to me in my profession *[behavior]*	Strongly disagree/agree
Beliefs about capabilities	Health professionals’ perceptions of facilitators and barriers to adopting the behavior	I am confident that I could *[behavior]* if I wanted to	Strongly disagree/agree
		For me, *[behavior]* would be. . .	Extremely difficult/easy
		I have the ability to *[behavior]*	Strongly disagree/agree
Moral Norm	Feeling of personal obligation regarding the adoption of the behavior	*[Behavior]* is the ethical thing to do.	Strongly disagree/agree
		It is acceptable to *[behavior]*	Strongly disagree/agree
Beliefs about consequences	Health professionals’ perception of the advantages and disadvantages that would result from behavior adoption	Overall, I think that for me *[behavior]* would be. . .	Useless/Useful
		Overall, I think that for me *[behavior]* would be. . .	Harmful/ Beneficial

Finally, the Personal Action Plan (PAP I&II) (Supplementary File 2), adapted from the Commitment to Change tool ( Shersheneva *et al.,* 2010), was implemented at two points in the study. The first part (PAP-I) was completed immediately after the workshop where participants listed: 1) anticipated goals in their teaching or clinical practice; 2) potential challenges; 3) ways to overcome those challenges and concrete steps to reach those goals. Six months post-intervention, participants completed the second part (PAP-II) assessing a) their confidence level b) perceived effectiveness of the workshop c) implementation level of anticipated goals, barriers and enablers, d) external resources accessed during the 6-month period post workshop. To facilitate the completion of PAP-II, participants received their previous PAP-I responses by email. The PAP is the only outcome measure reported that makes the distinction between participants’ type of practice (teaching or clinical).

### Data Analysis

Data was collected longitudinally over a period of three years. Quantitative data was analyzed with descriptive statistics using statistical software (SPSS version 24.0). Two coders independently analyzed all qualitative data (open-ended questions in PAP) by type of practice (i.e., teaching or practicing) using a direct content analysis approach ( Hsieh and Shannon, 2005). The qualitative analysis was guided by an adaptation of Theoretical Domains Framework (TDF) ( Michie *et al.,* 2005), where three categories (i.e. communication [under skills domain], teaching [under social influences domain] and appraisal/evaluation/review [under behavioral regulation domain]) were added. Coding discrepancies were discussed until a consensus was reached.

## Results/Analysis

Following the needs assessment results, findings are presented by the outcomes of participation, satisfaction, usefulness, knowledge and confidence levels, intention to change behaviour and reported changes in practice.

### Needs Assessment

Prior to each workshop, all participants completed an online needs assessment survey so as to tailor the intervention to their perceived needs. Findings indicate that despite targeting to a variety of audiences, similarities were found in the identified barriers and educational needs ( Table 5). Acquiring more knowledge on PS, QI and team communication were common perceived needs. Time constraints was a major barrier to implement PS-QI in the workplace, while role modeling was among the preferred teaching strategies.

**Table 5.** Results of three needs assessment surveys

Items	W1 (PS principles)	W2 (Communication)	W3 (QI)
**Demographic composition**	60 Program and site directors	Physicians (19 FPS & specialists) and 14 Allied Healthcare professionals	19 Specialists4 Family Physicians38 Allied Healthcare professionals2 managers1 med student
**Major barriers**	[Lack of] knowledge; Lack of resources (i.e. time); Organizational climate/culture	Lack of resources (i.e. time); [Lack of] knowledge	Environmental stressors (busy schedules); [Lack of] knowledge (Lack of familiarity with available resources);Environmental stressors (time restrictions).
**Preferred teaching strategy**	Role modeling	Role modeling	Role modeling and small group case-based discussions.
**Learners’ top educational needs**	Adverse events disclosure & prevention.	Access to Knowledge &Resources/Experiential Training	Knowledge and awareness of quality improvement principles; Exposure to quality improvement practices.

### CPD intervention: Participation outcome

Sixty-seven of 154 (43.5%) eligible HCPs from the fields of family medicine, physical and occupational therapy, nursing and other medical specialties participated in the study ( Table 6).

**Table 6.** Participation rate and demographics by specialization

Workshop	Participants in workshops	Research participants	Percent	Top Practice Areas
Workshop I	31	16	24%	Pediatrics (13%), anesthesiology (13%), family medicine (13%), psychiatry (13%)
Workshop I Repetition	23	14	21%	Nursing (29%), physical & occupational therapy (21%), communication sciences & disorders (14%)
Workshop II	45	16	24%	Nursing (31%), physical & occupational therapy (19%), communication sciences & disorders (13%), psychology (13%)
Workshop III	55	21	31%	Nursing (21%), quality evaluators (21%), oncology (11%), pediatrics (5%), family medicine (5%), physical & occupational therapy (5%)
**Total**	**154**	**67**	**100%**	

### CPD intervention: Satisfaction, Knowledge, Confidence Outcomes

Satisfaction was assessed directly (by asking how satisfied they were with the workshops) and indirectly (by asking the likelihood they would recommend the workshops to their peers). Satisfaction was high across all workshops and 65/67 (94%) of participants reported they would recommend the workshop to their colleagues.

**Figure 1.**
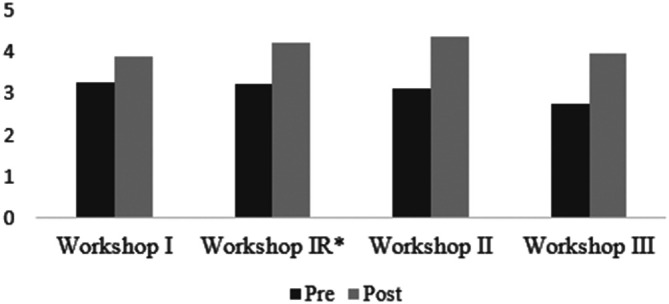
Post-Workshop Retrospective Pre-Post Questionnaire

* Workshop I (repeated)

**Figure 2.**
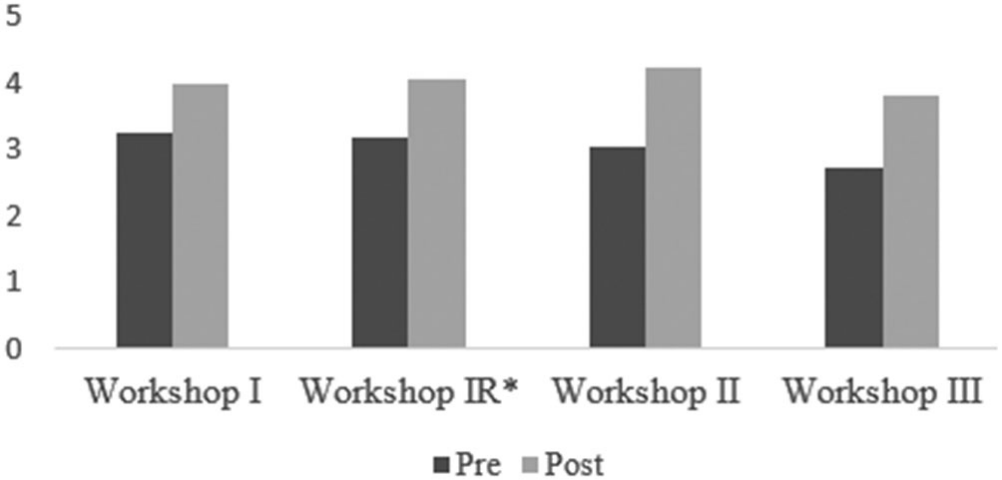
Post-Workshop Retrospective Pre-Post Questionnaire

A paired samples t-test confirmed a statistically significant difference between pre and post workshop knowledge levels for all items assessed. Overall, participants reported significant increase in knowledge ( figure 1) and confidence ( figure 2) levels post-intervention.

* Workshop I (repeated)

**Table 7.** Post-Workshop Retrospective Pre-Post Questionnaire: Knowledge and confidence differentials

	I have knowledge about...(highest differential)	I have confidence in my ability to ...(highest differential)	I have knowledge about ...(lowest differential)	I have confidence in my ability to ....(lowest differential)
**Workshop I**	How to instruct learners when adverse events occur(1.01)	Recognize personal factors that may influence patient safety (0.94)	How to enhance coping skills when faced with errors(-0.04)	Recognise systems factors that may influence patient safety (0.62)
**Workshop I (repeated)**	How to disclose adverse events to patients(1.49)	Disclose adverse events to patients(1.23)	Key patient safety concepts and processes (0.69)	Reflect on the impact of errors on self (0.54)
**Workshop II**	How to apply graded assertions(2.33)	Apply graded assertions(0.88)	Key leadership tasks(0.72)	Apply key components of effective teamwork communication that improve patient safety" (0.74)
**Workshop III**	Teaching tools resources(1.57)	Discuss QI strategies within the context of my practice (1.18)	How to overcome the barriers to implementing QI in my practice (1)	Overcome the barriers to implementing QI in my practice (0.81)

The gap analysis results between pre and post questionnaires across workshops are presented in table 7. Higher differentials indicate highest gaps in knowledge and confidence. Conversely, lowest differentials indicate highest levels of knowledge and confidence. In some instances, participants’ levels of knowledge and confidence follow similar patterns i.e., in WI (R), participants were less knowledgeable and less confident in the disclosure of adverse events to patients. Whereas in workshop III, they were knowledgeable and confident on “how to overcome the barriers to implementing QI in my practice”.

### CPD intervention: Reported performance outcome

Analysis of means scores of the CPD-Reaction questionnaire indicates that participants exhibited high scores on all five constructs of intention to change behaviour. Across workshops ‘social influence’ was consistently rated as the least influential construct in intention to change behaviour, while ‘moral norm’ (WI-WIR) and ‘beliefs about consequences’ (WII-III) were rated as the most influential ( Table 8).

**Table 8.** Post-workshop mean scores on the five constructs of the CPD-Reaction questionnaire

Construct	Workshop I	Workshop I (R)	Workshop II	Workshop III
Intention	6.22	6.12	6.45	5.54
Social Influence	**4.82**	**4.16**	**5.00**	**4.35**
Beliefs about capabilities	5.60	5.41	5.85	4.62
Moral Norm	**6.50**	**6.45**	6.41	5.94
Beliefs about consequences	6.38	6.12	**6.50**	**6.06**

### Personal Action Plan (I)

In PAP-I, participants stated two goals as well as anticipated barriers and enablers that might influence the implementation of those goals in their practice. Out of the 61 goals mentioned by clinical teachers (CT) and 64 clinical practitioners (CP), only the top five are discussed below.

The most frequently cited goal by CTs referred to teaching methods and techniques while CPs aimed to increase appraisals/evaluations/reviews to monitor their current practice. On the other hand, both CTs ( Table 9) and CPs ( Table 10) selected the same four out five goals which included: improving/increasing communication with staff and colleagues, increasing their knowledge and awareness on patient safety, applying the patient safety concepts learned to their practice and building teamwork to promote PS in hospital setting.

Similarly, both CTs and CPs mentioned the same top three barriers that may limit their success in implementing their PS & QI goals. These barriers included: lack of resources (i.e. time) (32/105), environmental stressors (e.g. heavy workload, multiple demands, conflicting roles) (28/105) and the organizational climate/culture (7/105).

In terms of enablers to overcome these challenges, CTs emphasized the importance of support from external resources (17/59) (e.g. formal training, teamwork, support from colleagues, management and champions), while CPs relied primarily on adopting/improving personal skills and procedures (e.g. performing more appraisals/evaluations, gaining a sense of empowerment to implement PS practices) and increasing their communication skills with team members and colleagues (9/38).

**Table 9.** Most frequently reported goals by Clinical Teachers in the Personal Action Plan (I)

		What goals do I have for teaching patient safety & QI?	What challenges may limit my success?	Who or what could help me overcome these challenges?	What concrete steps will I take to reach my goals?
1.	**Construct**	Teaching techniques, tools, methods (19/61)			
	**Examples**	*To incorporate teaching patient safety in resident program; Become more explicit about safety competencies in my teaching*	*Training educators who may not support the no-blame culture; changing curriculum*	*Work on educating others and finding teaching strategies to incorporate content; reorganize what is taught in my courses* *Role-modelling*	*Integrate a discussion on patient on a routine basis; Add patient safety to orientation of residents, try simulations on ward; Sessions with residents on adverse events*
2.	**Construct**	Communication skills (10/61)			
	**Examples**	*Improve team communication* *Role model communication* *Enhance communication skills*	-	*Discuss with contacts; discuss with the other members of our leadership team to incorporate in existing activities*	*Make explicit in daily discussions; Share my experience; Foster ongoing communication with SBAR during report & shift handover*
3.	**Construct**	[Increasing] knowledge/ Awareness (6/61)			
	**Examples**	*Increase staff awareness regarding patient safety* *Increase staff knowledge of SBAR*	*My knowledge base is limited* *Expertise on topic* *Knowledge*	*More education; having students/residents having heard that this is important before I mention it*	*Will prepare for students*
4.	**Construct**	Practice/skills development (6/61)			
	**Examples**	*Continuing to incorporate into my practice* *To make it integral to my team’s practice*	*Continual ongoing use* *Integrate KT activities*	*Practice in different settings* *Using SBAR tool*	*Start using tools in daily activities and interactions* *Start by incorporating many of the simple strategies learned today*
5.	**Construct**	Team working (5/61)			
	**Examples**	*How to work more collaboratively* *Improving teamwork*	*Ensuring cohesion*	*Working with others in same position* *Tools to help me engage the team* *Open minded members of team*	*Interdepartmental collaboration;* *Begin discussions on RTC and among teachers/* *Directors*

**Table 10.** Most frequently reported goals by Clinical Practitioners in the Personal Action Plan

		What goals do I have for practicing patient safety & QI?	What challenges may limit my success?	Who or what could help me overcome these challenges?	What concrete steps will I take to reach my goals?
1.	**Construct**	[Improve] communication (14/64)			
	**Examples**	*Improve disclosure* *Improve communication among staff and team members* *Being transparent to expose the risks*	*teaching in a way the patient understands*	*Discuss issues* *Exposing my recommendations on safety with patients in considering their individual culture/ personality and challenges*	*discuss with nurses, trainees, allied health professionals;* *listen;* *Empower trainee residents to speak freely and ask questions*
2.	**Construct**	Appraisal/evaluation/ review behaviour regulation (10/64)			
	**Examples**	*Reviewing of existing protocols* *Establishing processes* *Improving reporting to diminish errors*	*Broad aspect of compliance* *Whether or not is a real problem*	*Focused documentation* *Choose small goals that are achievable and don’t cost a lot of $$*	-
3.	**Construct**	Practice/skill development (5/64)			
	**Examples**	*Apply the practices learned* *Incorporate what learned to my practice* *Use & apply the SBAR*	-	*Start by incorporating many of the simple strategies learned today; [more] experience (junior); Training*	*Apply techniques learned* *Apply that in my practice on daily basis*find together how to incorporate in practice
4.	**Construct**	[Increase] knowledge (5/64)			
	**Examples**	*Will seek activities that increases my knowledge* *Awareness and expertise in patient safety concepts* *Will read more*	-	*More education*	*Report about the workshop;* *Prevention + “depistage” of all risks in initial Pt evaluation;* *Share QI /safety content with my colleagues*
5.	**Construct**	[Build] Teamwork (4/64)			
	**Examples**	*Effective handover* *Better team-building with briefing at beginning of each service period to clarify expectations* *Good team structure*	-	*Other healthcare professionals I am working with* *Engaging other team members to share concerns* *Open minded members of team, will be able to make the necessary changes*	*Actively involve the team by explaining my experience* *Start a team to improve procedure*

Triangulation of sources indicated similarities between the CPD-Reaction questionnaire goals which were derived from the intervention’s learning objectives (i.e., communication and teamwork in WI-WII; QI strategies in WIII) and the goals chosen independently by participants in the PAP-I (i.e., teaching methods, appraisals/evaluations/reviews; communication and teamwork). In other words, the CPD intervention’s content influenced the participant’s intention to apply it to their practice.

### Personal Action Plan (II)

Twenty-three percent (12/53) of participants across all workshops (except W1-R) completed the six-month follow-up measures ( Table 11). Due to the low response rate to the PAP-II, findings presented henceforth have been combined across all workshops.

**Table 11.** Personal Action Plan II: Response rate

	n/N	%
Workshop I	4/16	25
Workshop II	6/16	37.5
Workshop III	2/21	9.52

On average, participants reported higher confidence levels immediately following the workshop, and a slight decline 6-month post intervention ( Table 12).

**Table 12.** Personal Action Plan II: Mean confidence levels over W1-W3

	Pre *	Post *	6-month follow-up **
WI-WIII	2.99	4.02	3.90

* pre and post, n= 24

** 6 months, n = 12

In terms of goal attainment, participants reported partially implementing 18/23 (78%) of their anticipated goals ( Table 13).

**Table 13.** Personal Action Plan II: Levels of goal implementation

	Fully achieved	Partially achieved	Not achieved
Goal 1	2	9	1
Goal 2 *	1	9	1
**Total**	**3**	**18**	**2**

* 1 missing answer

Accessing resources (i.e., more time, workshops, and literature) (10/29), conducting more reviews/evaluations (4/29), teamwork (3/29) and social support (2/29) were among the top five frequently reported enablers that enhanced goal implementation (PAP-II). Some participants (4/10) cited the CPD intervention as the most useful resource in helping them achieve their PS & QI goals, as it provided them with increased knowledge and a sense of empowerment to advocate for patient safety. These results are consistent with the needs assessment, where lack of knowledge was one of the main identified barriers. Additional resources/support that would help to achieve anticipated goals included management commitment, communication with staff and colleagues and being motivated to implement PS concepts learned ( Table 14). Conversely, the most commonly cited barriers dealt with limited resources (e.g., time constraints and work overload) (7/25) followed by the type of organizational climate/culture (4/25). These results matched the needs assessment findings where lack of resources (i.e. time), environmental stressors (busy schedules) and organizational culture were the major barriers identified by participants.

**Table 14.** Six-Months Post-Workshop Responses to PAP-II

		What has helped you achieve your goals?	What barriers limited your success to achieve the first & second goal?	What additional resources/support would help you achieve your first & second anticipated goal?
1	**Construct**	Resources/material resources ( availability and management) (10/68)		
	**Examples**	*The workshop help me incorporate patient’ safety principles* *Embark on patient safety projects* *Participation on unit rounds, workshops*	*Time constraints (x6)* *Poor technical environment*	*More time* *Literature about safety in the workspace* *More workshops* *Mentorship*
2	**Construct**	Social support (7/68)		
	**Examples**	*Mutual support when unit overloaded to assure security of patients* *Collaboration* *Stakeholders to help support and facilitate change*	*Lack of hospital-to-hospital partnerships to improving patient safety and quality of care*	*Want to start a patient experience group* *Communication with patients*
3	**Construct**	Team working (7/68)		
	**Examples**	*I feel empowered to improve teamwork* *Collaborative team discussions* *Restructure of the tasks between team members*	-	*Collaboration* *Concerted effort by a team or colleague* *Teamwork*
4	**Construct**	Communication (with staff, colleagues) (7/68)		
	**Examples**	*Increased communication and use of SBAR w/ staff and colleagues* *Bringing back discussions to patient safety issues* *Communication to other colleagues*	**-**	*communication and mutual respect between health care providers* *communication*
5	**Construct**	Appraisal/evaluation review (6/68)		
	**Examples**	*Following audit results* *Achieving consistency of care between health care providers* *Review the existing principles on QI taught*	*Ability to assess sustainability*	*A person to help collect for the action plan implementation and to monitor results* *Auditing* *Formal process of review*

## Discussion

This study showed the feasibility to develop and implement an effective CPD intervention targeting healthcare professionals’ knowledge and confidence on how to teach and practice PS-QI. The intervention’s effectiveness was assessed via the outcomes of participation, usefulness, satisfaction, knowledge, confidence and reported performance.

Across the intervention, levels of satisfaction and usefulness were high and significant increase in knowledge and confidence were reported immediately post-intervention. Significant acquisition of knowledge and confidence mirrored the results of a systematic review on QI ( Boonyasai *et al.,* 2007), multifaceted interventions on team communication ( Roman, Abraham and Dever, 2016), a team-based QI leadership training ( Rao *et al.,* 2016), a program on clinicians’ use of physical restraints ( Chang *et al.,* 2016), and residents’ training on the use of ionizing radiation ( Sheng *et al.,* 2016). In our study, confidence levels slightly decreased after 6 months. Future studies should use effective reinforcement strategies such as checklists, facilitated debriefing, telephone interviews, coaching ( Neily *et al.,* 2010), hands-on instruction ( Potts, Shields and Upshur, 2016) and spaced education testing (SE) ( Larsen, Butler and Iii, 2008; Kerfoot and Baker, 2012; Bruckel *et al.,* 2016).

Overall, the attendees’ intentions to change their practice is consistent with the PS-QI literature. Our participants rated *beliefs about consequences* and *moral norm* as the most influential factors to implement PS-QI behaviors that mirror a study on HCP’s error reporting ( Wallace *et al.,* 2009). Conversely, *social influence* was the least influential factor, implying that participants would apply the PS-QI behaviors regardless of the approval or disapproval of significant others in the workplace. These results contradict the PAP-I findings where social support and teamwork were among the most cited enablers, but is consistent with the PS-QI literature. For instance, studies targeting healthcare professionals in different contexts rated high the influence of social norm ( Wallace *et al.,* 2009), role modeling of professional peer behavior ( Wakefield *et al.,* 2010; White *et al.,* 2015) and implementing Root Cause Analysis (RCA), while White et al. found that nurses considered colleagues and supervisors as the most significant referents to support for hand hygiene practice ( White et al., 2015).

The present intervention focused on team communication, which was identified by participants as a goal to implement in their practice and is key to achieving a safety culture ( Kirk *et al.,* 2007). Team communication in the workplace has been a gap in physicians’ traditional training which has primarily emphasized the autonomy-centered medical expert role and which might be a barrier to engage physicians in the collaborator and communicator roles within an interprofessional team ( Botwinick, Bisognano and Haraden, 2006). Staff training in PS-QI has been identified as one of the five resources included in economic evaluations of healthcare systems ( Tompa *et al.,* 2016).

Formal training was one of the main anticipated enablers which was implemented 6 month post-intervention. Our findings are consistent with a systematic review that depicted successful training as dependent on the curriculum, instructional strategies and organizational variables such as leadership support, resource availability, training environment and readiness for change ( Lo *et al.,* 2011). An 8-hour crisis intervention course in psychiatry indicated that staff education, senior leadership monitoring and use of reporting resulted in positive impact on practice ( Blair *et al.,* 2016). However, providing training on effective team communication ( Velji *et al.,* 2008) might be insufficient to achieve a safety culture ( Wakefield *et al.,* 2010). In fact, participants’ anticipated systemic barriers to implement PS-QI behaviours included lack of resources, environmental stressors and the need to decreasing the punitive culture on reporting incidents and accidents ( Wakefield *et al.,* 2010). Ginsburg et al. (2012) found that new graduates in medicine, nursing and pharmacy reported increased confidence in PS learning related to effective communication, particularly in a clinical setting.This finding contrasts that of our own, wherein participants from workshop II reported the lowest confidence on teamwork communication. An effective strategy to improve PS in primary care is incident reporting and analysis which is enhanced by a non-blaming culture ( Verstappen *et al.,* 2015). An interactive workshop was effective in changing nurses’ beliefs and attitudes ( Potylycki *et al.,* 2006) from a punitive to an open, non-blaming culture.

The intervention was effective in supporting reported impact on practice six month post-intervention where enablers involved access to resources, social support, team working and communication. These results are in line with models that acknowledge the integration of both the individual and the system level of responsibility to support patient safety ( Makary and Daniel, 2016). A safety culture requires strong leadership and organizational support, open and safe environments to report and discuss adverse events with effective on-going reporting systems, QI and training ( Canadian Patient Safety Institute, 2018; Verstappen *et al.,* 2015; Forster *et al.,* 2011; Goldstein *et al.,* 2017; Scott *et al.,* 2003). On the other hand, barriers to fully implement anticipated goals in practice included lack of resources, environmental stressors, organizational climate/culture, and lack of social support. These findings are consistent with PS-QI interventions in a variety of contexts such as HCPs conducting RCA ( Wallace *et al.,* 2009), adopting surgical checklist in the OR ( Stevens *et al.,* 2011), nurses increasing hand hygiene practice ( White *et al.,* 2015) and physicians providing physical activity advice to reduce blood pressure ( Presseau *et al.,* 2009).

A systematic analysis indicated that poor organisational culture, inadequate infrastructure, and system shocks characterize organizations struggling to implement PS-QI ( Vaughn *et al.,* 2018). Examples of system shocks are substantial healthcare system reforms such as the 2015 reform in Quebec which introduced an extensive reorganization of healthcare institutions, the centralization of all healthcare services and the imposition of patient quotas to general practitioners under the threats of punitive and coercive measures ( Gore, 2017; Fidelman, 2017). Unsurprisingly, this type of environmental stressor might have influenced the implementation of the anticipated goals six month-post intervention. However, more research is needed to fully examine the impact of the healthcare reform in Quebec.

The three-party partnership supported the feasibility of this study. Breaking the silos amongst CPD, Faculty Development and a teaching hospital was an effective strategy to involve key stakeholders ( Roman, Abraham and Dever, 2016) in order to start building a culture of continuous improvement ( Sargeant, Wong and Campbell, 2018), sharing tools to be used by frontline users ( Chen *et al.,* 2014), and focusing on learner, faculty and organizational factors ( Wong *et al.,* 2010; Canadian Association of School of Nursing and Canadian Patient Safety Institute, 2018). To ensure continuity and effectiveness of the intervention, this integrated approach should continue to be driven by the *learning healthcare system* that emphasizes workplace learning, the development of communities of practice ( Davis, Rayburn and Smith, 2017) and the alignment of professional development (micro-pathway) with health system leaders (macro-pathway) to support the Canadian health system reform ( Davis and Rayburn, 2016).

### Limitations

There are several limitations with the present study. Firstly, due to the absence of a control group the impact of the educational intervention could have been influenced by other contextual variables. Secondly, the self-reported nature of the measures used is susceptible to social desirability bias and recall bias from participants ( Althubaiti, 2016) as well as healthcare professionals’ limited self-assessment ability ( Davis *et al.,* 2006). Future studies could triangulate self-reported questionnaires with more objective measures such as chart audits ( Donnellan, Sweetman and Shelley, 2013). Thirdly, the high attrition rate (50%-80%) during the 6-month follow-up reduced the sample size and the generalizability of our findings. Furthermore, a small budget limited the selection of strategies to increase the effectiveness of the CPD intervention such as simulation ( Gardner *et al.,* 2016). Lastly, the recruitment of physicians was a challenge. Iterations of the intervention will diversify recruitment strategies by involving leadership support, emphasizing the medical expert and scholar roles and enlarging the target audience to include residents.

## Conclusion

Patient safety and quality improvement are pillars to enable the health care reform worldwide. CPD supports healthcare professionals’ life-long learning and provides opportunities for improving competence and performance and ultimately, patient care and population health. This CPD intervention provided effective training to healthcare professionals in the province of Quebec. Its impact on participants’ practice was mediated by dynamic, organizational culture and subcultures. Lessons learned contributed to the emerging field integrating CPD with PS-QI.

## Take Home Messages

• Multifaceted, theory-driven CPD interventions are effective strategies to increase participants’ knowledge, confidence and to apply principles of patient safety and quality improvement.
• Moral norm and beliefs about consequences were the most important factors influencing participants’ intention to change behavior. Future interventions should build case-based discussions around ethical and moral issues.
• Implementing a three-party partnership with key stakeholders supported the feasibility of this intervention and the building of a safe culture.
• Interprofessional representation on the planning committee contributed to the successful development and dissemination of the CPD intervention tailored to the needs of the target audience.

## Notes On Contributors

**FRANCESCA LUCONI**, PhD, is the Assistant Dean and Academic Associate of Continuing Professional Development at McGill University. As a researcher and instructional designer, she has experience in the development and evaluation of professional training programs in healthcare. Her research interests include patient safety/QI, e-CME, lifelong learning, metacognition, cognitive psychology and instructional technology.

**MIRIAM BOILLAT**, MDCM, CCFP, FCFP, is Associate Dean of Faculty Development and Associate Professor of Family Medicine. Dr. Boillat practices and teaches family medicine at St. Mary’s Hospital. Her educational interests include undergraduate medical education (in particular medical interviewing and communication skills), postgraduate medical education, and faculty development.

**SUSANNE MAK**, MSc, OT(c), erg. is an occupational therapist, an Assistant Professor (professional) and Associate Director of the Occupational Therapy program (School of Physical and Occupational Therapy). Her research interests pertain to the phenomena of attrition and retention in the rehabilitation professions, mentoring and professional identity.

**DANIEL CHARTRAND**, MD PhD FRCPC, is the Vice-Chairman of the Department of Anesthesia at McGill University and the Co-Chair of the Patient Safety Committee of the Federation of Specialist Physicians of Quebec. Since 2001, he has been involved in several patient safety initiatives at the national and international levels.

**NADINE KORAH**, MD, is an Attending Physician in the Division of General Pediatrics and the Assistant Program Head of the Medical Inpatient Services at the Montreal Children’s Hospital, McGill University Health Center. She obtained her MSc in Quality Improvement and Patient Safety from the University of Toronto. The focus of her academic career includes QI research, integration of patient safety into medical and post-graduate education, participating in continuing medical education workshops around Patient Safety, and organizing in-situ simulations on the general pediatric wards.

**MARK DALY**, MA (Ed.), is the Patient Safety Lead - Postgraduate Medical Education, Assistant Professor and Director of Faculty Development for Interprofessional Education at McGill University. Mark collaborates with internal and external partners to develop and deliver patient safety initiatives focusing on interprofessional collaboration, communication and creating a culture of patient safety.

**MERON TEFERRA**, MSc, is the Research Assistant of Continuing Professional Development at McGill University. She obtained her MSc in Health Psychology from the University of Bath. Her research interests include medical education, treatment adherence, positive psychology, and motivation.

**JENNIFER GUTBERG**, MSc, is a PhD Candidate at the Institute of Health Policy, Management, and Evaluation at the University of Toronto, and a Research Fellow with the Health System Performance Research Network. Her research explores healthcare delivery from an organizational lens, with interests in patient safety, culture, integrated care, and leadership.
