# Supplementary material for: Patient Safety and Quality of Care are Everybody's Business: Evaluating the Impact of a Continuing Professional Development Program beyond Satisfaction
Source: MedEdPublish (2016). 2019 Mar 11;8:46. [Version 1] doi: 10.15694/mep.2019.000046.1 (PMC10712615; doi:10.15694/mep.2019.000046.1)
Supplement: Peer review history

# Reviewer report: Reviewer response for version 1

**DOI:** 10.21956/mep.19791.r29757

**Published:** 2019-07-02

**Authors:** Ken Masters

**Affiliations:** 1 Sultan Qaboos University

This review has been migrated. The reviewer awarded 5 stars out of 5 An interesting paper on evaluating the impact of a CPD program on patient safety. The authors have clearly identified the problem of Patient Safety in healthcare, and the Introduction goes into substantial detail about the different approaches to CPD training, in order to give a solid theoretical basis for the CPD training outlined in this study.The study has been extremely well conducted and, as is given in the title, goes beyond the normal evaluation of workshops and looks at the impact of the training in far more detail, including later reported performance. The authors have also spent considerable time discussing their methodology and results in light of the literature, While the attrition rate (and the resulting impact on the sample size) is a concern, this does not impact negatively on the paper enough to detract from its overall value. The conclusion tends to sell the paper rather short, but, overall, it remains a fine paper, and a fine example of workshop running and evaluation.Well done, indeed.

Reviewer Expertise:

NA

No decision status is available

# Reviewer report: Reviewer response for version 1

**DOI:** 10.21956/mep.19791.r29758

**Published:** 2019-03-28

**Authors:** Helena Filipe

**Affiliations:** 1 Hospital of the Armed Forces/PL-EMGFA

This review has been migrated. The reviewer awarded 5 stars out of 5 Luconi and associates focus their research on a relevant and increasing attention subtheme in CPD: patient safety (PS) and quality improvement (QI). They studied the impact of a learning intervention consisting of three workshops in improving knowledge on PS and QI by studying satisfaction, usefulness, knowledge, confidence, intention to change behavior and reported changes in practice. They thoroughly elaborate on the introduction section and provide adequate evidence to substantiate their writing. The methodology used is clearly described and illustrated by tables well positioned in the text to facilitate the reader.The section of results is long and present several comparisons among the studied variables. It would be interesting to see in the future the impact in performance beyond the commitments to change intentions reported and in a next study finding the use of additional evaluation tools that can provide an objective input.The manuscript highlights a relevant topic, describes an investigation extensively designed, judiciously informed by educational theory with very promising results.The Authors exhaustively discuss their findings and frame them in the literature reviewed taking care to point out their study limitations but also open a pathway to continue investigation in this field. We can have the conviction that good teaching of any topic will result in knowledge improvement if the learner is interested and engages. Taking this to the CPD level can offer additional challenges when compared to other medical education levels, especially when a prominent systems’ based component is involved. In their research, Luconi and colleagues proved this can happen. Congratulations.

Reviewer Expertise:

NA

No decision status is available
